# Andrographolide emeliorates maltol aluminium-induced neurotoxicity via regulating p62-mediated Keap1-Nrf2 pathways in PC12 cells

**DOI:** 10.1080/13880209.2021.1883678

**Published:** 2021-02-26

**Authors:** Jiaqi Lu, Lili Gu, Qin Li, Ningzi Wu, Hongxing Li, Xinyue Zhang

**Affiliations:** Laboratory of Neuropsychiatric Drug Research of Zhejiang Province, Hangzhou medical college, Hangzhou, P.R. China

**Keywords:** Neuroprotective, anti-inflammation, autophagy

## Abstract

**Context:**

Andrographolide (Andro) has a neuroprotective effect and a potential for treating Alzheimer’s disease (AD), but the mechanism has not been elucidated.

**Objective:**

The efficacy of Andro on p62-mediated Kelch-like ECH-associated protein 1(Keap1)-Nuclear factor E2 related factor 2 (Nrf2) pathways in the aluminium maltolate (Al(mal)_3_)-induced neurotoxicity in PC12 cell was explored.

**Materials and methods:**

PC12 cells were induced by Al(mal)_3_ (700 μM) to establish a neurotoxicity model. Following Andro (1.25, 2.5, 5, 10, 20, 40 μM) co-treatment with Al(Mal)_3_, cell viability was detected with MTT, protein expression levels of β-amyloid precursor protein (APP), β-site APP cleaving enzyme 1 (BACE1), Tau, Nrf2, Keap1, p62 and LC3 were measured via western blotting or immunofluorescence analyses. *Nrf2, Keap1, p62* and *LC3* mRNA, were detected by reverse transcription-quantitative PCR.

**Results:**

Compared with the 700 μM Al(mal)_3_ group, Andro (5, 10 μM) significantly increased Al(mal)3-induced cell viability from 67.4% to 91.9% and 91.2%, respectively, and decreased the expression of APP, BACE1 and Keap1 proteins and the ratio of P-Tau to Tau (from 2.75- fold to 1.94- and 1.70-fold, 2.12-fold to 1.77- and 1.56-fold, 0.68-fold to 0.51- and 0.55-fold, 1.45-fold to 0.82- and 0.91-fold, respectively), increased the protein expression of Nrf2, p62 and the ratio of LC3-II/LC3-I (from 0.67-fold to 0.93- and 0.94-fold, 0.64-fold to 0.88- and 0.87-fold, 0.51-fold to 0.63- and 0.79-fold, respectively), as well as the mRNA expression of *Nrf2, p62* and *LC3* (from 0.48-fold to 0.92-fold, 0.49-fold to 0.92-fold, 0.25-fold to 0.38-fold). Furthermore, Nrf2 and p62 nuclear translocation were increased and keap1 in the cytoplasm was decreased in the presence of Andro. Silencing *p62* or *Nrf2* can significantly reduce the protein and mRNA expression of Nrf2 and p62 under co-treatment with Andro and Al(mal)_3_.

**Discussion and conclusions:**

Our results suggested that Andro could be a promising therapeutic lead against Al-induced neurotoxicity by regulating p62-mediated keap1-Nrf2 pathways.

## Introduction

Alzheimer’s disease (AD) is a serious neurodegenerative disease with a high incidence in the elderly and the feature that incidence increases with increased age. The major pathological features are plaques formed by extracellular β-amyloid protein (amyloid-β, Aβ) deposition (Farzi et al. 2018) and tangles of nerve fibres formed by intracellular hyperphosphorylated Tau protein. The imbalance between the production and removal of misfolded proteins (Aβ and Tau) produces a cascade effect, which aggravates the process of AD through oxidative stress, inflammation and neuron apoptosis. The nuclear factor (erythroid-derived 2)-like 2 (Nrf2)-Kelch-like ECH-associated protein 1 (Keap1) pathway is one of the main cellular defense mechanisms against oxidative stress. Nrf2 can induce transcription factors and regulate various cellular antioxidant systems including oxidative stress, protein toxicity, metabolic stress and inflammation. SQSTM1/p62, a substrate of autophagy, can identify proteins that need to be degraded (Peng et al. 2017). In AD, the formation and transportation of autophagosomes are damaged, which will lead to the disorder of autophagy function, thus increasing the level of harmful proteins and aggravating the pathological features of AD (Wang et al. 2019; Wan et al. 2019). Importantly, there is a connection between Nrf2-keap1 and p62. P62 can recruit Keap1 from the Nrf2-Keap1 complex, sequester Keap1 into autophagosomes, make Nrf2 translocate to the nucleus and regulate antioxidative stress genes (Yang et al. 2019). In short, p62 promotes Nrf2 nuclear translocation, so the p62-Nrf2-Keap1 pathway plays a critical role in AD.

Andrographolide (Andro), present in the medicinal plant *Andrographis paniculata* (Burm.f.) Nees (Acanthaceae), shows a variety of pharmacological activities including antibacterial (Banerjee et al. 2017), anti-inflammation (Gupta et al. 2020), and immune regulation. Recently it has been found related to the treatment of AD (Cisternas et al. 2019; Xu et al. 2019; Lindsay et al. 2020). Andro can ameliorate the cognitive impairment through the Wnt signalling pathway and glycogen synthase kinase 3β (GSK3β) inactivation, reduce neuro-inflammation and oxidative stress (Seo et al. 2017; Yang et al. 2017), restore the length and function of synapses and stimulate neurogenesis (Varela-Nallar et al. 2015). Our previous study found that Andro could significantly resist Aβ-induced cytotoxicity in PC12 cells and was related to the activation of Nrf2 and p62 (Gu et al. 2018).

Aluminium (Al) is neurotoxin and accumulates in the brain gradually with age, causing oxidative brain damage related to diseases such as AD (Song 2018; Lu et al. 2020). Al-maltolate [Al(mal)_3_] treatment simulated AD-like neuropathology which emerged Aβ deposition, neurofibrillary tangles, apoptosis, and oxidative stress in hippocampus, forebrain, and midbrain regions. However, to date, not report is available to demonstrate the neuroprotective role of Andro on p62-Nrf2-Keap1 pathways in PC12 cell lines following Al(mal)_3_ exposure. Therefore, in the present study, the neurotoxicity induced by Al(mal)_3_ in PC12 cells was established, then β-amyloid precursor protein (APP), β-site APP cleaving enzyme 1 (BACE1) and Tau protein level, Nrf2-Keap1 and p62 expression along the effects of Andro treatment during Al toxicity were analysed.

## Materials and methods

### Chemicals and cell culture

Andro (purity 98%), obtained from Yuanye bio-technology company (Shanghai, China), presents as a white powder dissolved in dimethyl sulfoxide (DMSO, Sigma Shanghai, China), then diluted in the culture medium. Aluminium chloride hexahydrate (97% purity) and maltol (99% purity) were purchased from Aladdin (Shanghai, China).

Highly differentiated PC12 cells were purchased from the cell centre of the Chinese academy of medical sciences (Beijing, China) and cultured in Dulbecco’s modified Eagle’s medium (DMEM, Gibco, Gaithersburg, MD, USA) with 10% foetal bovine serum (FBS) and 0.1% penicillin/streptomycin at 37 °C in a humidified atmosphere with 5% CO_2_.

### Preparation of Al(mal)_3_

The preparation method referred to the literature (Dhivya Bharathi et al. 2019). Crystalline aluminium chloride was prepared into a solution of 20 mM PBS. Maltol was dissolved in the preheated 0.1 M PBS to a final concentration of 60 mM, and then the two solutions were mixed by the same volume to prepare Al(mal)_3_ solution with concentration of 10 mM, pH was adjusted to 7.2 ∼ 7.4 by dropwise addition of 10 N NaOH and a 0.22 µm filter was used for extraction. Al(mal)_3_ solution of 10 mM was used as the mother liquor and diluted into different concentrations of Al(mal)_3_ solution by using complete medium of corresponding volume, respectively.

### MTT assay

The cell viability was detected by MTT assay. Firstly, the cells were plated in a 96-well plate at a density of 1 × 10^5^ cells/mL, with 200 μL per well. Cells were divided into normal, Al(mal)_3_ treatment and Al(mal)_3_ + Andro co-treatment groups. Medium containing a certain concentration of Andro or Al(mal)_3_ was added into each well in a volume of 100 μL for 24 h. Then 20 μL of 5 mg/mL MTT solution was added into each well. After cells were incubated at 37 °C for 4 h. DMSO (150 μL) was further added after discarding the culture medium. The optical density was measured at 570 nm in Cytation 1 imaging reader (BioTek, USA).

### Transient gene silencing by small interfering RNAs (siRNAs)

Cells were inoculated into a 6-well plate for 24 h before transfection, and then replace with 2 mL fresh culture medium (containing serum, without antibiotics). siRNA (100 pmol) and 5 µL Lipo 6000TM transfection reagent was put into two EP tubes containing 125 µL DMEM, respectively. After mixing and standing for 5 min, transfection reagent and siRNA was mixed together and incubated at room temperature for 20 min. Mixture (250 μL) per well was put evenly into the entire well in the 6-well plate and replaced with normal culture medium after 6 h. The siRNA duplexes were synthesized and sequences for the siRNA were as follows: Nrf2 siRNA: 5′-GAAGCUCAGCUUGCAUUAA-3′ (sense), 5′-UUAAUGCAAGCUGAGCUUC-3′ (antisense); p62 siRNA: 5′-UAUCAGUUGUACUAAUCCCUU-3′ (sense), 5′- GGGAUUAGUACAACUGAUAGU-3′ (antisense).

### Western blotting analysis

Cells were inoculated into a 6-well plate and divided into normal, Al(mal)_3_ treatment, Andro treatment and Al(mal)_3_+Andro co-treatment groups. Total proteins were extracted by RIPA lysis buffer and quantified by BCA protein assay kit (ThermoFisher, USA). Each sample (20 μg) was separated by 12% SDS-PAGE and then transferred onto polyvinylidene difluoride membranes (Millipore, USA). The membranes were then blocked with 5% (w/v) fat-free milk for 1 h, followed by incubation overnight at 4 °C with primary antibodies at 1:1000 dilution ratio: APP(1007-5, huaanbio, China), BACE1(5606, CST, USA), p-Tau (Ser396) (AF3148, Affinity, USA), Tau (AF1249, Beyotime, Shanghai, China), Keap1 (sc-514914, Santa Cruz, USA) Nrf2 (ab62352, Abcam, USA), p62 (AF5384, Affinity, USA), LC3 (bs-8878R, Bioss, China), GAPDH (FD0063, Fudebio, China). After washing, the membranes were incubated with horseradish peroxidase (HRP)-conjugated secondary antibodies for 1 h. The membranes were rinsed in the western lighting plus-ECL solutions, and finally the immunoreactive bands were detected using Chemiluminescence Imaging System (ChemiScope 3000 Mini, Clinx, China). The relative optical density of the digitized image was analysed by Image J software.

### Immunofluorescence assay

Cells was fixed with 4% paraformaldehyde for 15 min, washed with PBS, and add 0.2% Triton X-100 (9002-93-1, Sigma, USA) for 10 min. After washed with PBS, cells were blocked with 5% block BSA for 2 h, incubated with primary antibody: Nrf2 (1:200), keap1 (1:500) or p62 (1:200) overnight at 4 °C. Then washed with PBS, cells were incubated with FITC-labeled goat anti-rabbit (A0562, Beyotime, Shanghai, China) or Cy3-labeled goat anti-mouse IgG (H + L) (A0521, Beyotime, Shanghai, China) (1:200), and put in a dark room at room temperature for 2 h. Finally, antifade mounting medium with DAPI (P0131, Beyotime, China) was added to the slide, and the image was photographed by Cytation 1 imaging reader.

### Reverse transcription quantitative polymerase chain reaction (RT-qPCR)

Total RNAs were extracted from cells by using the RNAprep pure cell/bacteria kit (Tiangen, China), and reverse transcribed into cDNA by Fasting gDNA Dispelling RT SuperMix (Tiangen, China). The RT-qPCR analysis was performed using ABI 7500 fast system (Applied Biosystems, USA) with SuperReal PreMix Colour (SYBR Green) (Tiangen, China). The primers were synthetized from Sangon Biotech (Shanghai, China) and shown as follows: β-actin: 5′-GCAGGAGTACGATGAGTCCG-3′ (forward), 5′-ACGCAGCTCAGTAACAGTCC-3′ (reverse); p62: 5′-GTCAATTTCCTGAAGAATGTGGG-3′ (forward), 5′-GAGTTCACCTGTGG ATGGGTC-3′ (reverse); LC3: 5′-TTCTGGTCAGGTTCTCCCCA-3′ (forward), 5′-CCCAGGACTTGGTATGCTGG-3′ (reverse); Nrf2: 5′-GCCCTCAGCATGATGGACTT-3′ (forward) and 5′-GTTTGGGAATGTGGGCAACC-3′ (reverse); Keap1: 5′-TGGGTCAAATACGACTGCCC-3′ (forward) and 5′- TGGCTCATATCTCTCCACGC-3′ (reverse); Melting curve data were analysed to determine PCR specificity. Relative fold expressions were analysed using the 2^–ΔΔCt^ method and using β-actin Ct values as the internal reference in each sample.

### Statistical analysis

Statistical analysis was performed using GraphPad prism 5.0 statistical software (San Diego, CA, USA). All data are presented as mean ± standard error of the mean (SEM). Differences between two groups were assessed using Student’s *t*-test, and one-way analysis of variance (ANOVA) was used to assess differences between multiple groups, with *p* < 0.05 considered statistically significant. All the experiments were performed in triplicate.

## Results

### Effect of Andro on the cell viability in Al(mal)_3_-treated PC12 cells

**Figure 1.**
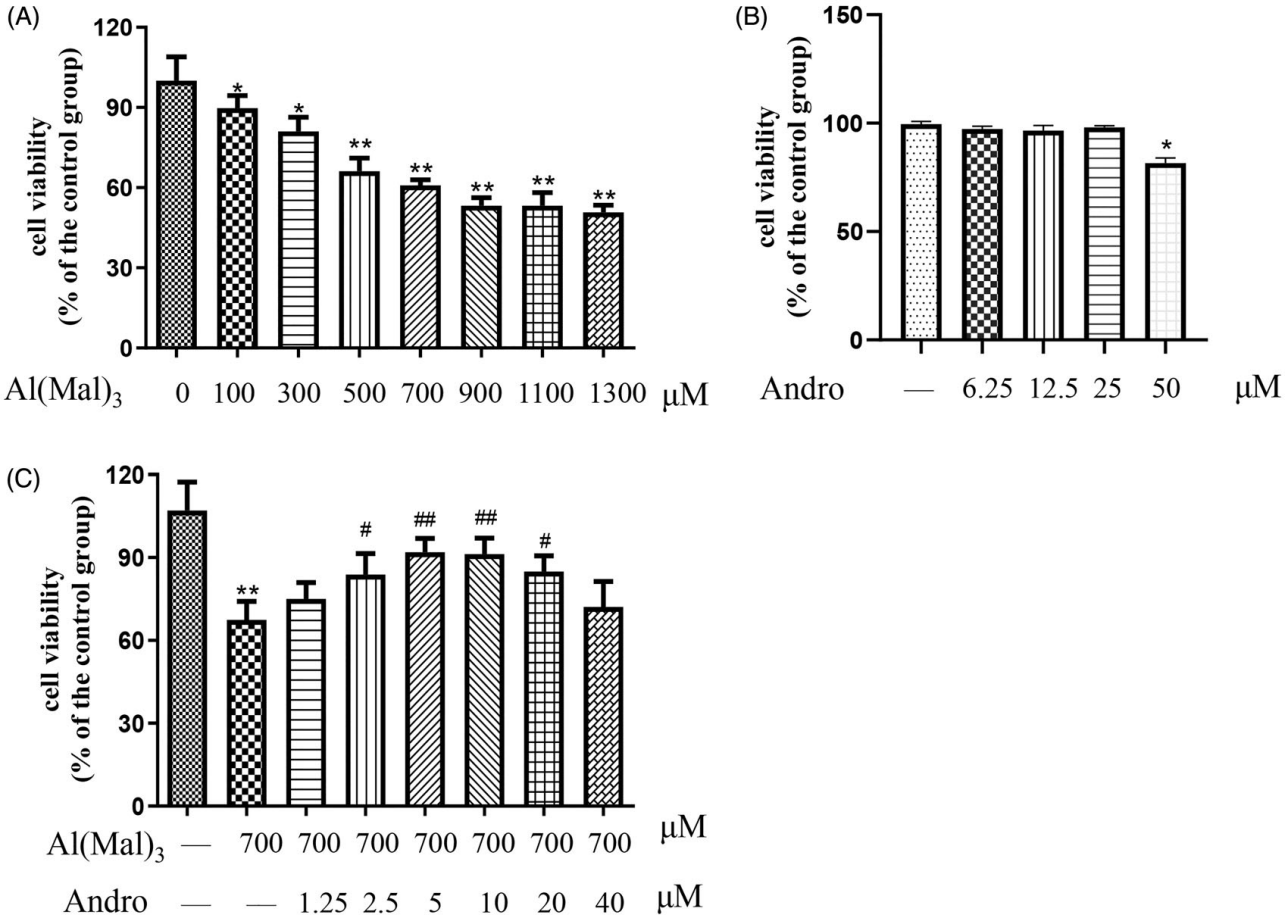
Andro ameliorated Al(mal)_3_-induced toxicity in PC12 cells. Cells were treated with different concentrations of Al(mal)_3_ (100, 200, 400, 600, 800, 1200 and 1600 μM) for 24 h (A); Cells were treated with different concentrations of Andro (6.25, 12.5, 25, 50 μM) for 24 h (B); Cells were co-treated with 700 μM Al(mal)_3_ and different concentrations of Andro (1.25, 2.5, 5, 10, 20 and 40 μM) for 24 h (C). Cell viability was measured by MTT assay (*n* = 6) *p < 0.05, **p < 0.01 versus the control. ^#^*p* < 0.05, ^##^*p* < 0.01 versus Al(mal)_3_ group was considered statistically significant differences.

Firstly, the cytotoxicity of Al(mal)_3_ on PC12 cells was detected by MTT assay. As shown in Figure 1(A), after treated with different concentrations of Al(mal)_3_ in cells for 24 h, the cell viability was decreased (*p* < 0.05, *p* < 0.01). The IC_50_ value was 1182 μM and 95% confidence intervals were 1036–1348 μM. The cell viability with Al(mal)_3_ 700 μM treatment was about 60%, and there was no significant change when dose was greater than 700 μM under our experimental conditions. Here 700 μM Al(mal)_3_ was selected as the modelling concentration. Results in Figure 1(B) showed PC12 cells were not damaged after treated by different concentrations of Al(mal)_3_ below 25 μM for 24 h. As shown in Figure 1(C), after co-treated with different concentrations of Andro and 700 μM Al(mal)_3_ in cells for 24 h, the cell viability in 2.5, 5,10 and 20 μM Andro groups were significantly increased compared with the model group (*p* < 0.05, *p* < 0.01), which suggested that Andro alleviated Al(mal)_3_-induced cell death. In addition, the protective effect of Andro decreases at 20 and 40 μM, suggesting high therapeutic concentrations are not necessarily effective and may have an inhibitory effect on cells. Andro 5 and 10 μM were selected as effective drug concentrations for follow-up experiments.

### Effect of Andro on the protein expression of APP and BACE1 in Al(mal)_3_-induced PC12 cells

**Figure 2.**
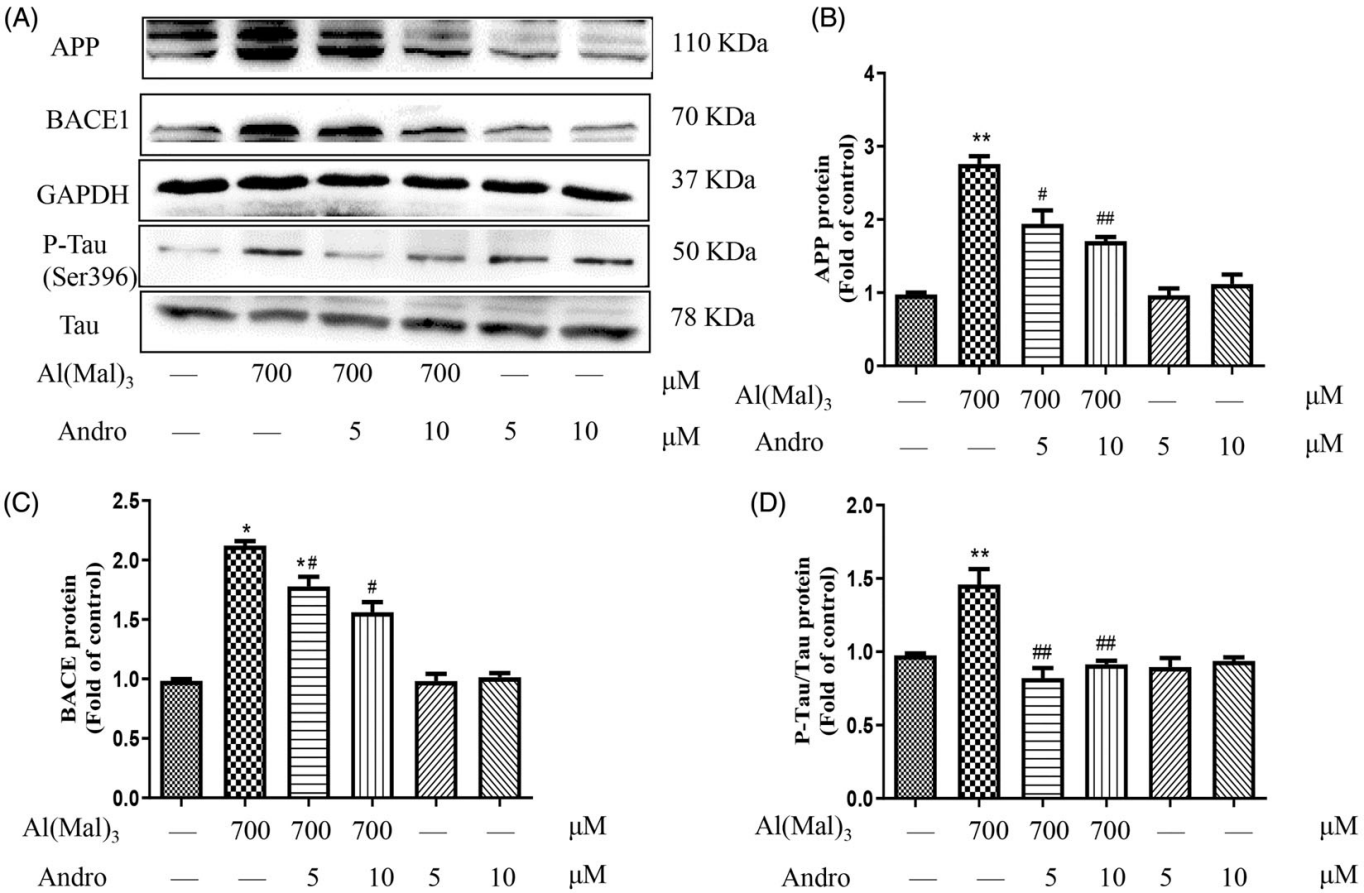
Andro inhibited the expression of APP, BACE1 and p-Tau in PC12 cells induced by Al(mal)_3_. Cells were incubated with 700 μM Al(mal)_3_ and 5 or 10 μM Andro for 24 h. The protein expression of APP, BACE1, p-Tau (ser396) and Tau were detected by western blot, GAPDH was used as loading control (A); Quantitative analysis of APP (B), BACE (C) and p-Tau/Tau (D) protein expression levels; *p < 0.05, **p < 0.01 versus the control, ^#^*p* < 0.05, ^##^*p* < 0.01 versus Al(mal)_3_ group was considered statistically significant differences (*n* = 3).

APP can be cleaved by BACE1 and produce the toxic Aβ oligomers, which were typical pathological features of AD with Tau hyperphosphorylation. As shown in Figure 2(A,B), compared with the normal control group, the levels of APP, BACE1, and p-Tau proteins in the Al(mal)_3_ group was increased significantly (*p* < 0.05, *p* < 0.01), indicating Al(mal)_3_ could accelerate the production of toxic Aβ oligomers and Tau phosphorylation. Compared with the Al(mal)_3_ group, Andro 5 and 10 μM significantly suppressed the up-regulation of APP, BACE1 and p-Tau induced by Al(mal)_3_ treatment (Figure 2(B–D)) (*p* < 0.05, *p* < 0.01), which suggested that Andro intervene in Aβ formation and Tau proteins phosphorylation to alleviate Al(mal)_3_-induced cell damage.

### Effect of Andro on the protein expression of Nrf2 and Keap1 in Al(mal)_3_-induced PC12 cells

**Figure 3.**
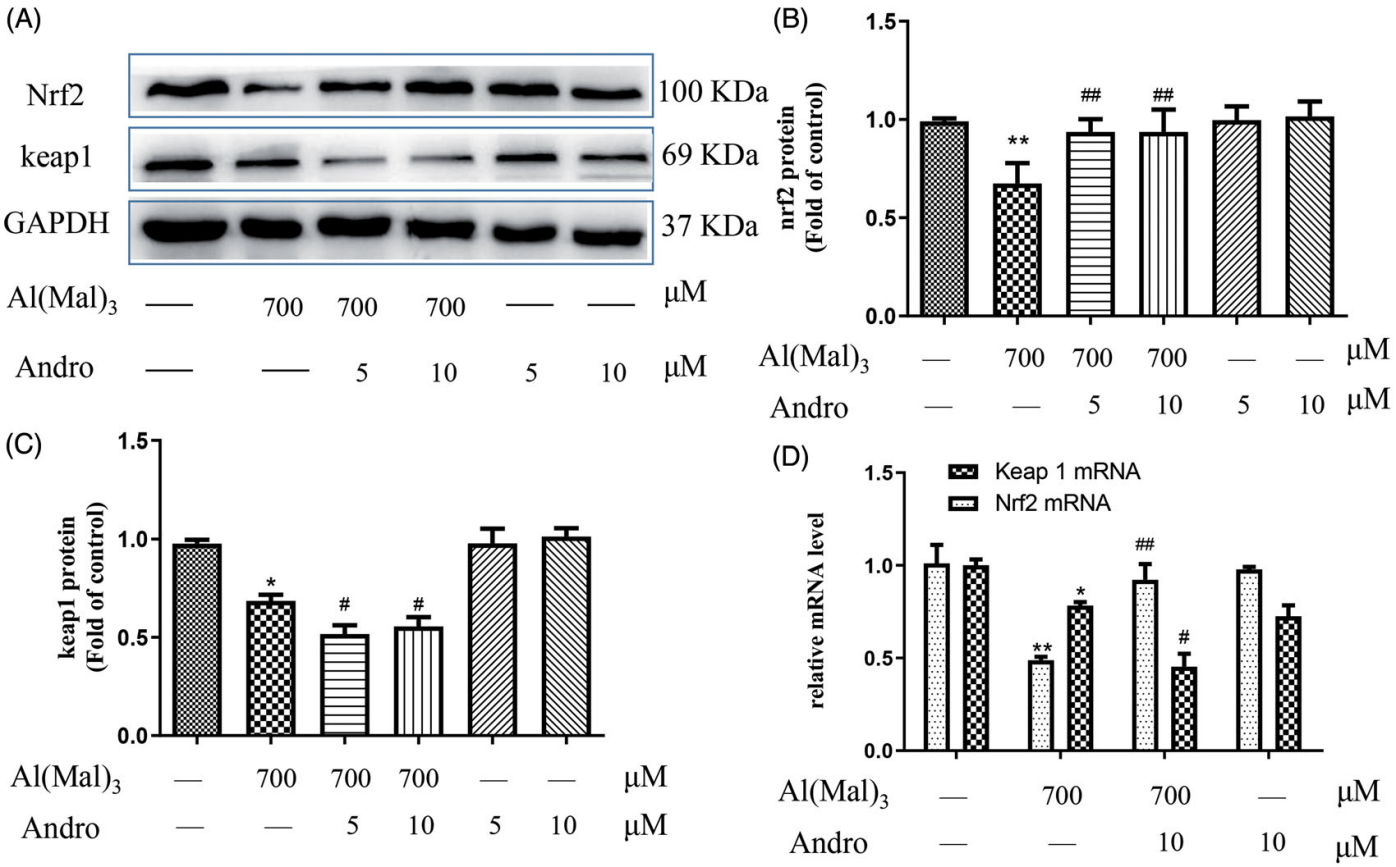
Andro enhanced on the expression of Nrf2 and Keap1 proteins and mRNA in PC12 cells induced by Al(mal)_3_. Cells were incubated with 700 μM Al(mal)_3_ and 5 or 10 μM Andro for 24 h. The expression of Nrf2 and Keap1 were detected by western blot, GAPDH was used as loading control (A); Quantitative analysis of Nrf2 (B) and Keap1 (C) protein expression levels; The levels of Nrf2 and Keap1 (D) mRNA were analysed using RT-qPCR. *p < 0.05, **p < 0.01 versus the control, ^#^*p* < 0.05, ^##^*p* < 0.01 versus Al(mal)_3_ group was considered statistically significant differences (*n* = 3).

Keap1-Nrf2 system activation is the major oxidative stress response pathway. Keap1, an adaptor of the ubiquitin ligase complex that targets Nrf2, plays a major role in Nrf2 degradation. As shown in Figure 3, the expression of Nrf2 and Keap1 protein in the Al(mal)_3_ group had a significant decrease compared with the normal control group (*p* < 0.01). Andro 5 or 10 μM up-regulated the Nrf2 protein expression (Figure 3(B)) and down-regulated the Keap1 in Al(mal)_3_-treated cells (Figure 3(C)) (*p* < 0.05) which were consistent with mRNA levels from PCR results (Figure 3(D)).

**Figure 4.**
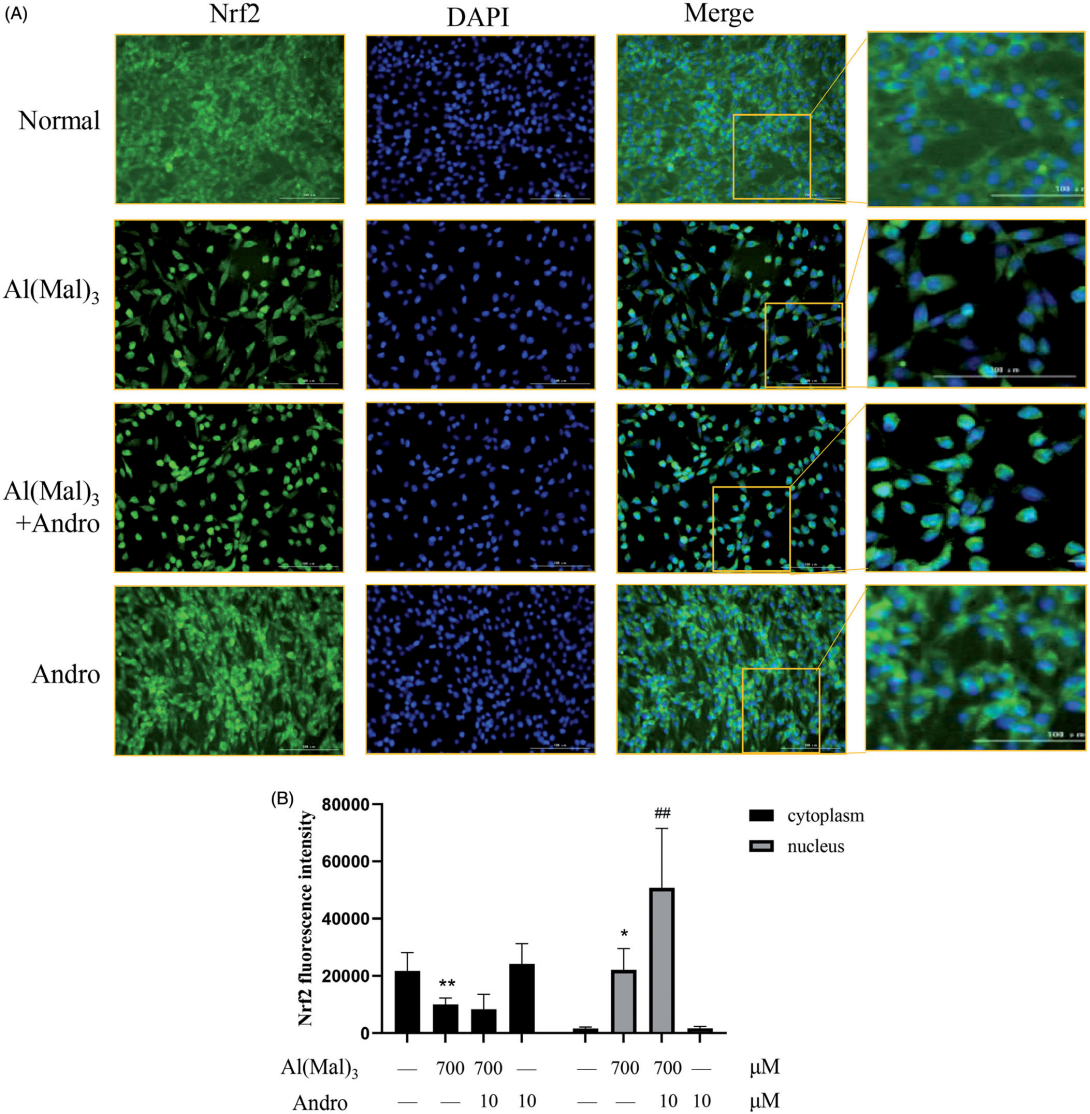
Andro increased the nuclear expression of Nrf2 in Al(mal)_3_-induced PC12 cells. Cells were co-incubated with 700 μM Al(mal)_3_ and 10 μM Andro for 24 h. The fluorescence localization of Nrf2 was measured with immunofluorescence. An anti-Nrf2 antibody was used to detect Nrf2 localization using a fluorescence microscope. Green colour represented FITC-positive Nrf2, Blue colour represented DAPI-positive nucleus. Bar = 100 μm (A); Quantitative immunofluorescence analysis of Nrf2 in cytoplasm and the nucleus (B). *p < 0.05, **p < 0.01 versus the control, ^##^*p* < 0.01 versus Al(mal)_3_ group was considered statistically significant differences (*n* = 6).

To further verify Nrf2 activation induced by Andro treatment, immune fluorescence was used to observe the positional changes of intracellular Nrf2 protein. As shown in (Figure 4(A,B)), the fluorescence intensity of Nrf2 was in accordance with the western blotting results. The immunofluorescence staining results indicated that the fluorescence intensity of Nrf2 in the nucleus was decreased in Al(mal)_3_-induced cells. However, Andro and Al(mal)_3_ co-treatment enhanced the fluorescence intensity of Nrf2 in the nucleus and markedly reversed the abnormal nuclear translocation of Nrf2.

### Effect of Andro on the protein and mRNA expressions of p62 and LC3 in Al(mal)_3_-induced PC12 cells

**Figure 5.**
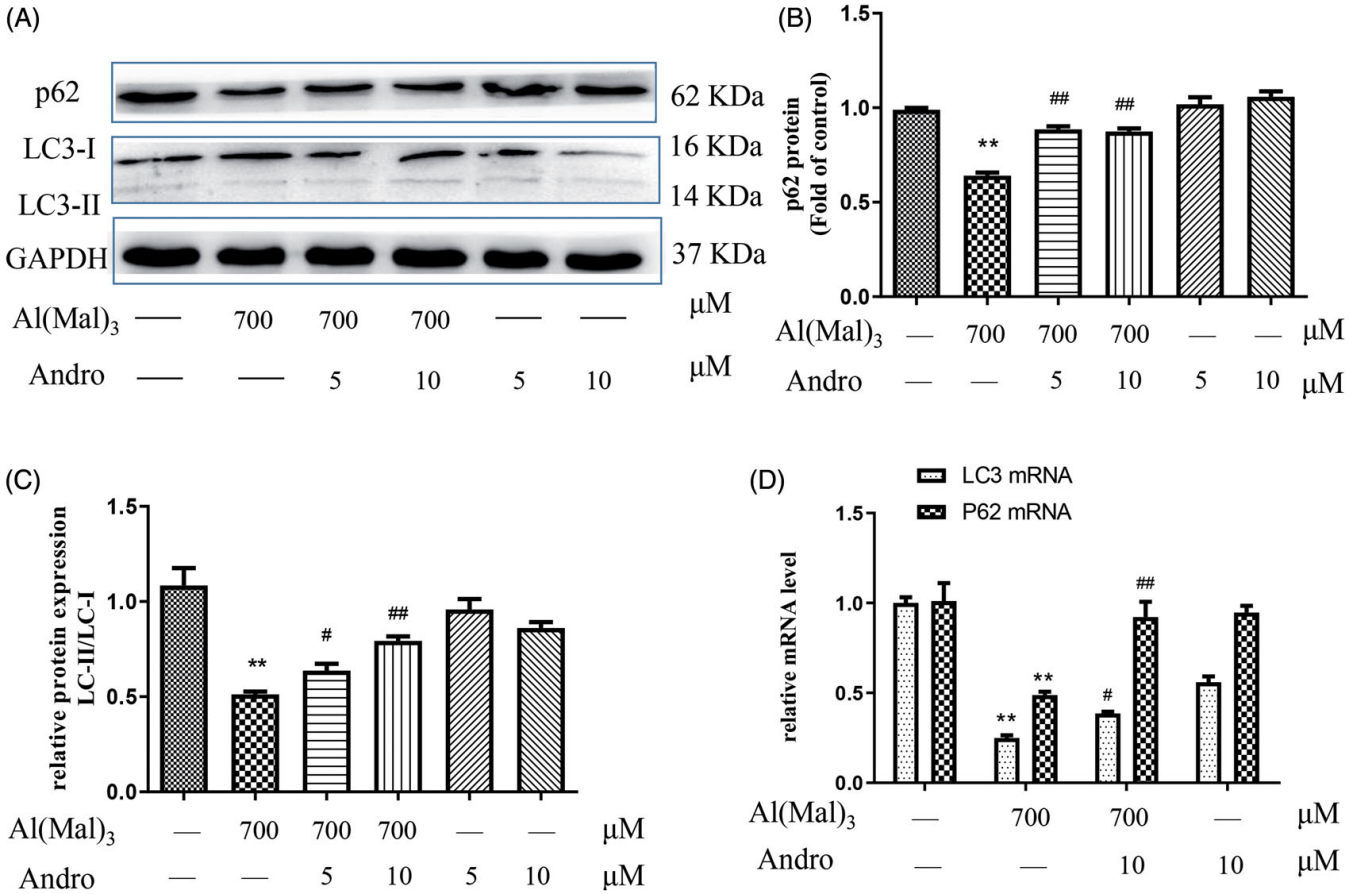
Andro upregulated the expression of p62 and LC3 proteins and mRNA in PC12 cells induced by Al(mal)_3_. Cells were incubated with 700 μM Al(mal)_3_ and 5 or 10 μM Andro for 24 h. The expression of p62 and LC3 proteins were detected by western blot, GAPDH was used as loading control (A). Quantitative analysis of p62 (B) and the ratio of LC3-II to LC3-I (C) protein expression levels. The levels of p62 and LC3 (D) mRNA were analysed using RT-qPCR *p < 0.05, **p < 0.01 versus the control, ^#^*p* < 0.05, ^##^*p* < 0.01 versus Al(mal)_3_ group was considered statistically significant differences (*n* = 3).

As shown in Figure 5, compared with the normal group, the expression of p62, a key autophagy adaptor and the ratio of LC3-II to LC3-I in the Al(mal)_3_ group was significantly reduced (*p* < 0.01), the expression of p62 and the ratio of LC3-II to LC3-I proteins in co-treated Al(mal)_3_ and Andro 5 or 10 μM group were higher than that of the Al(mal)_3_ group (Figure 5(B,C)) (*p* < 0.05, *p* < 0.01). The results of *p62* and *LC3* mRNA levels were shown in Figure 5(D). Compared with the normal group, Al(mal)_3_ triggered a significant decrease in *p62* and *LC3* mRNA (*p* < 0.01), while 10 μM Andro restored the levels of *p62* and *LC3* mRNA induced by Al(mal)_3_ (*p* < 0.05, *p* < 0.01).

### Effect of Andro on the location of p62 and keap1 proteins in Al(mal)_3_-induced PC12 cells

**Figure 6.**
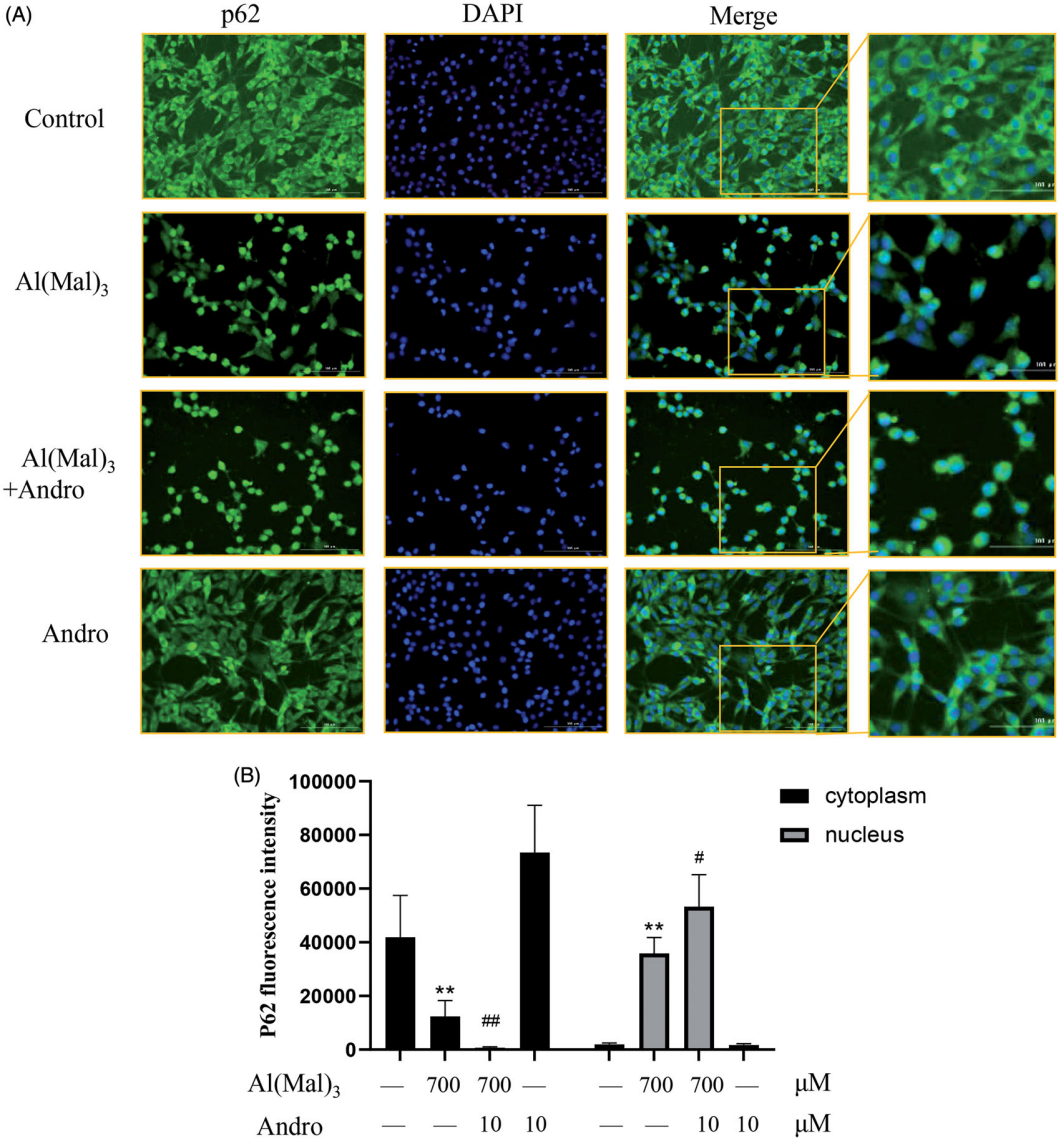
Effect of Andro on expression and location of p62 protein in PC12 cells induced by Al(mal)_3_. Cells were incubated with 700 μM Al(mal)_3_ and 10 μM Andro for 24 h. The fluorescence localization of p62 was measured with immunofluorescence. An anti-p62 antibody was used to detect p62 localization using a fluorescence microscope. Green colour represented FITC-positive p62, Blue colour represented DAPI-positive nucleus. Bar = 100 μm (A); Quantitative immunofluorescence analysis of p62 in cytoplasm and the nucleus (B). *p < 0.05, **p < 0.01 versus the control, ^#^*p* < 0.05, ^##^*p* < 0.01 versus Al(mal)_3_ group was considered statistically significant differences (*n* = 6).

**Figure 7.**
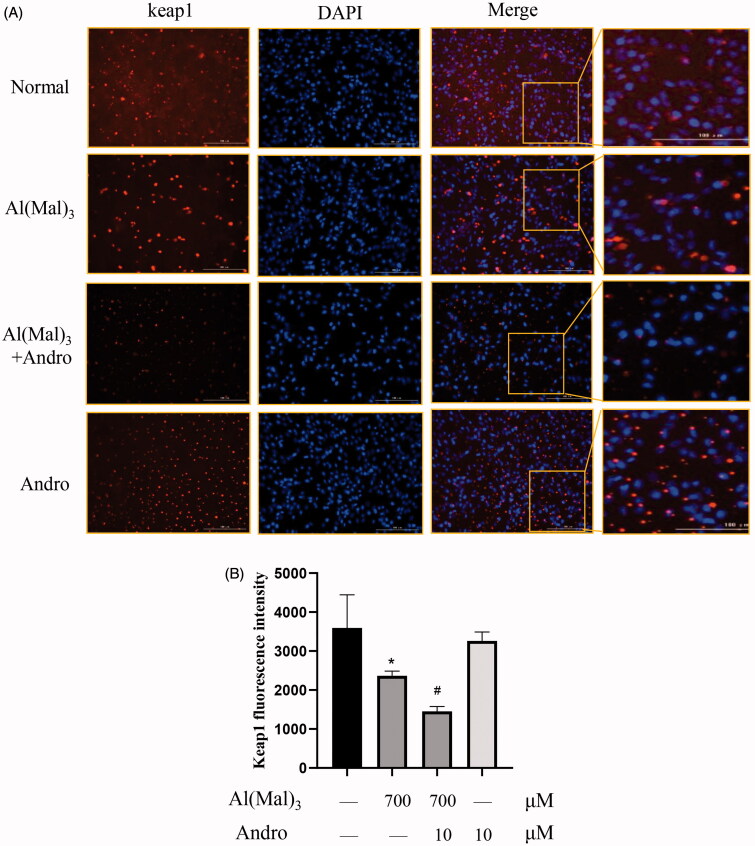
Effect of Andro on expression and location of keap1 protein in PC12 cells induced by Al(mal)_3_. Cells were incubated with 700 μM Al(mal)_3_ and 10 μM Andro for 24 h. The fluorescence localization of keap1 was measured with immunofluorescence. An anti-keap1 antibody was used to detect keap1 localization using a fluorescence microscope. Red colour represented Cy3-positive keap1, Blue colour represented DAPI-positive nucleus. Bar = 100 μm (A); Quantitative immunofluorescence analysis of Keap1 in cytoplasm (B). *p < 0.05 versus the control, ^#^*p* < 0.05 versus Al(mal)_3_ group was considered statistically significant differences (*n* = 6).

The immunofluorescence staining results indicated that compare with the normal group, the fluorescence intensity of p62 and keap1 in the cytoplasm and nucleus was decreased in Al(mal)_3_-induced cells. However, Andro and Al(mal)_3_ co-treatment augmented fluorescence intensity of p62 in the nucleus (Figure 6(A,B)) and reduced fluorescence intensity of keap1 in the cytoplasm (Figure 7(A,B)). The fluorescence intensity of p62 and keap1 were in accordance with the western blotting results.

### Effect of silencing p62 or Nrf2 on the protein and mRNA expression of p62, LC3 and Nrf2 in co-treated with Andro and Al(mal)_3_ PC12 cells

**Figure 8.**
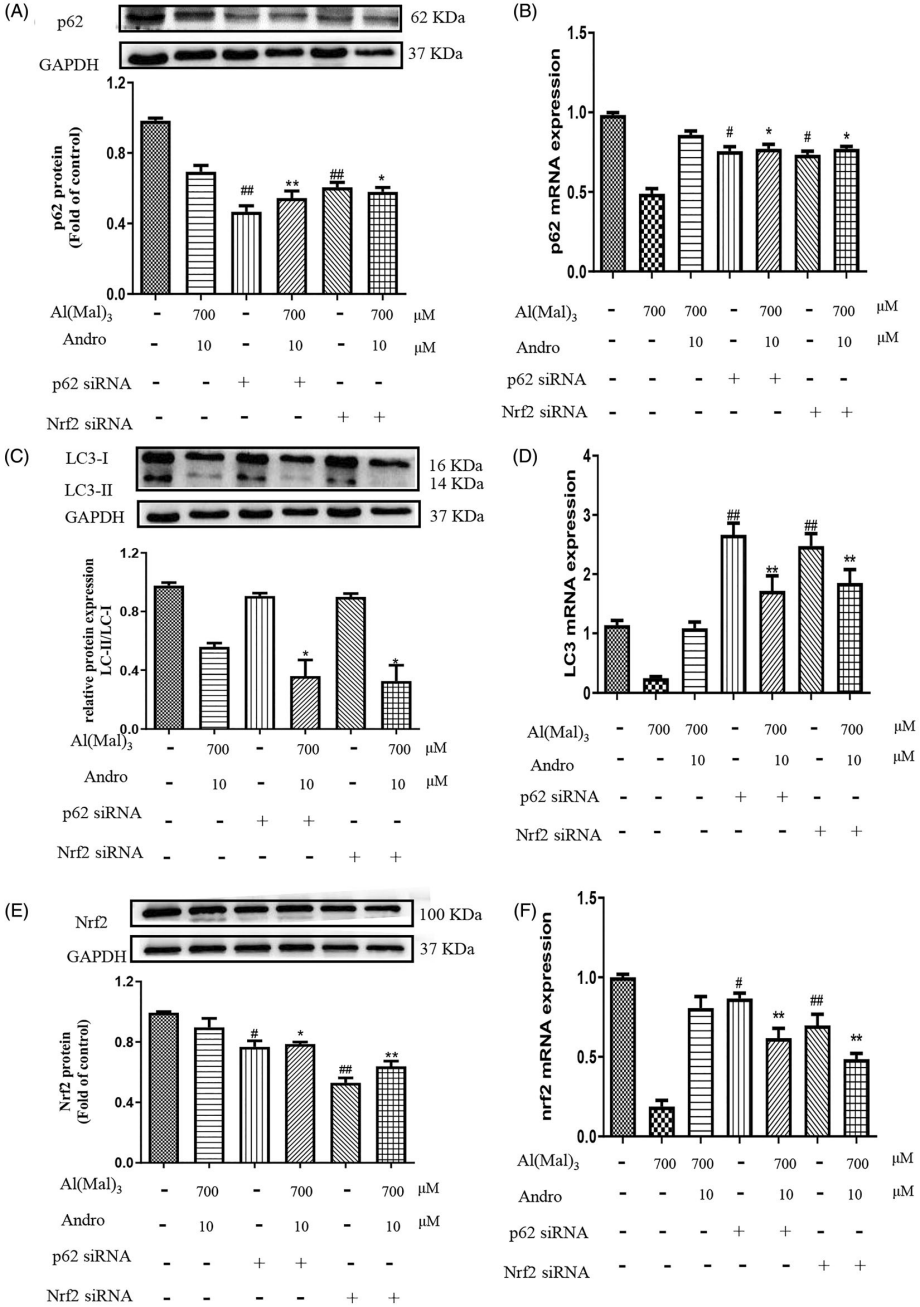
Effect of silencing p62 or Nrf2 on the expression of p62, LC3, Nrf2 proteins and mRNA in PC12 cells co-treated with Al(mal)_3_ and Andro. After transfection with Nrf2 or p62 siRNA, cells were incubated with 700 μM Al(mal)_3_ and 10 μM Andro for 24 h. The expression of p62 (A), LC3 (B) and Nrf2 (E) were detected by western blot, GAPDH was used as loading control. The levels of *p62* (B), *LC3* (C) and *Nrf2* (F) mRNA were analysed using RT-qPCR. ^#^*p* < 0.05, ^##^*p* < 0.01 versus the control, *p < 0.05, **p < 0.01 versus Al(mal)_3_ + Andro group was considered statistically significant differences (*n* = 3).

As shown in Figure 8, compare with the normal group, only p62 or Nrf2 siRNA treatment significantly reduced the protein and mRNA expression of p62 (Figure 8(A,B)) and Nrf2 (Figure 8(E,F)) (*p* < 0.05, *p* < 0.01), but didn’t change the ratio of LC3-II/LC3-I protein (Figure 8(C)) and increased *LC3* mRNA level (Figure 8(D)) (*p* < 0.01). Compare with co-treated with Andro and Al(mal)_3_ group, Nrf2 siRNA pre-treatment reduced the protein and mRNA expression of p62 (*p* < 0.05), p62 siRNA pre-treatment reduced the protein and mRNA expression of Nrf2 (*p* < 0.05, *p* < 0.01), Nrf2 or p62 siRNA pre-treatment deceased the ratio of LC3-II/LC3-I protein (*p* < 0.05) and increased LC3 mRNA level (*p* < 0.01). This lack of response suggests that Nrf2 or p62 siRNA treatment may suppress LC3-II protein expression through post-translation regulation.

## Discussion

It has been reported that neuroprotection of Andro in multiple cell models, including LPS-induced PC12 neurons, Aβ_(1–42)_-induced microglial and mix glial (Das et al. 2017; Seo et al. 2017; Yang et al. 2017; Xu et al. 2019). Aluminium has obvious neurotoxicity and is an important environmental risk factor leading to a variety of neurodegenerative diseases such as AD. Al(mal)_3_ could release a large amount of Al^3+^ under physiological pH conditions, making it an ideal material for the study of aluminium neurotoxicity (Johnson et al. 2005). Previous experiments from our lab indicated that the treatment of Andro (less than 50 µM) didn’t result in dominant cell death in normal PC12 cells (Gu et al. 2018). In this experiment, Al(mal)_3_ (700 μM) caused ∼ 60% of cell death in PC12 cells under our laboratory conditions, Andro at concentration of 5 and 10 μM obviously ameliorated Al(mal)_3_-induced cell death, preliminarily showing that Andro had neuroprotection in Al-induced injury.

Cleavage of APP by BACE-1 is the rate-limiting step in Aβ production and a neuropathological hallmark of AD (Peters et al. 2019). In our previous study, the network pharmacology method was used to reveal that the *Andrographis paniculata* acts on AD, which is involved in the APP/BACE/glycogen synthase kinase 3β (GSK3β) axis (Gu et al. 2020). In this experiment, the expression of APP and BACE1 proteins in the Al(mal)_3_ group was significantly increased compared with the normal group, speculating that Al(mal)_3_ may enhance the cleavage effect of BACE1 on APP to increase Aβ production. Andro significantly decreased the expression of APP and BACE1 proteins, hinting that Andro reduced the cleavage of APP and the formation of Aβ induced by Al(mal)_3_. Meanwhile, Andro treatment lowered the up-regulated of p-Tau protein, an important component of neurofibrillary tangles in Al(mal)_3_-treated cells.

Keap1 is a component of the cullin-3 ubiquitin ligase of Nrf2, which can mediate the degradation of Nrf2 as an inhibitor of Nrf2 (Wang et al. 2020). In normal physiology, Nrf2 binds to Keap1 and is degraded by the proteasome through ubiquitination. But under the oxidative stress induced by Aβ (Huang et al. 2020), the modification of Keap1 cysteine residues leads to the inhibition of Nrf2 ubiquitination, thereby stabilizing Nrf2, allowing Nrf2 to accumulate in the cytoplasm and then transfer to the nucleus to activate antioxidant genes (Zhang et al. 2019). In our experiment, Andro can regulate the Nrf2-Keap1 signalling pathway, confirmation of Nrf2 nuclear translocation was monitored by immunofluorescene.

P62, an indicator of autophagic degradative activity, has several functional domains, including the ubiquitin-binding domain and the LC3-interacting region (LIR). AD-like pathology could be improved by increasing p62-mediated selective autophagy (Caccamo et al. 2017), such as intracellular Aβ and over-expressed Tau. Autophagy also participates in the degradation of BACE1 and APP C-terminal fragment (Tian et al. 2013; Bera et al. 2020). LC3-I is a cytosolic Atg that during autophagosome formation is post-translationally modified to form LC3-II. P62 binds to LC3 to facilitate selective autophagy through its LIR domain, so the ratio of LC3-II to LC3-I combined with p62 protein can reflect the situation of autophagy. Our results showed that Al(mal)_3_ significantly reduced mRNA and protein expressions of p62 and the ratio of LC3-II to LC3-I, indicating that p62-dependent autophagy was inhibited. Co-treatment with Andro obviously reversed this phenomenon. It was speculated that Andro can ameliorate the inhibition of p62-mediated autophagy by Al(mal)_3_. As for the decrease of LC3 mRNA and protein caused by the 10 μM Andro control group, the reason may be that cells reduce the expression of LC3 protein by reducing the expression of LC3 mRNA to maintain autophagy homeostasis through negative feedback regulation, thus restoring the enhancement of autophagy by Andro.

Another important aspect is that p62 binds to Keap1 and promotes the degradation of Keap1, leading to the activation of Nrf2 (Wang et al. 2020). Therefore, the lack or overproduction of p62 in autophagy will affect the activation of Nrf2 (Komatsu et al. 2010). Immunofluorescence experiments also confirmed that Al(mal)_3_ and Andro co-treatment elevated the expression of p62 and reduced keap1. Therefore, Andro may also promote the degradation of Keap1 through p62 by increasing the expression of p62, thus activating the Nrf2 signalling pathway. Our results demonstrate that the silence of p62 can lead to a decrease in mRNA and protein level of Nrf2 under Al(mal)_3_ and Andro co-treatment, which may be due to the fact that p62 can regulate Nrf2 transcription and cause the degradation of Keap1. The silence of Nrf2 can also reduce p62 protein and mRNA expression, which was consist with the results from Santarino et al. (2017). Our results showed that there was a positive feedback loop in PC12 cells co-treated by Al(mal)_3_ and Andro, but how Nrf2 depends on which pathway to influence p62 deserves further study.

## Conclusions

The present study showed that Andro could ameliorate the damage of PC12 cells induced by Al(mal)_3_, decrease the expression of APP, BACE1 and p-Tau and increase the expression of p62 and LC3 and regulate the Nrf2-Keap1 pathways, and there is positive feedback between Nrf2-Keap1 and the p62 pathway. Our data showed that Andro could be a promising therapeutic lead against Al-induced neurotoxicity by regulating p62-mediated keap1-Nrf2 pathways, providing a basis for perfecting the mechanism of Andro in the treatment of AD.

## References

[CIT0001] Banerjee M, Parai D, Chattopadhyay S, Mukherjee SK. 2017. Andrographolide: antibacterial activity against common bacteria of human health concern and possible mechanism of action. Folia Microbiol. 62(3):237–244. 28097636 10.1007/s12223-017-0496-9

[CIT0002] Bera S, Camblor-Perujo S, Calleja Barca E, Negrete-Hurtado A, Racho J, De Bruyckere E, Wittich C, Ellrich N, Martins S, Adjaye J, et al. 2020. AP-2 reduces amyloidogenesis by promoting BACE1 trafficking and degradation in neurons. EMBO Rep. 21(6):e47954. 32323475 10.15252/embr.201947954 PMC7271323

[CIT0003] Caccamo A, Ferreira E, Branca C, Oddo S. 2017. p62 improves AD-like pathology by increasing autophagy. Mol Psychiatry. 22(6):865–873. 27573878 10.1038/mp.2016.139 PMC5479312

[CIT0004] Cisternas P, Oliva CA, Torres VI, Barrera DP, Inestrosa NC. 2019. Presymptomatic treatment with andrographolide improves brain metabolic markers and cognitive behavior in a model of early-onset Alzheimer’s disease. Front Cell Neurosci. 13:295–313. 31379502 10.3389/fncel.2019.00295 PMC6657419

[CIT0005] Das S, Mishra KP, Ganju L, Singh SB. 2017. Andrographolide – a promising therapeutic agent, negatively regulates glial cell derived neurodegeneration of prefrontal cortex, hippocampus and working memory impairment. J Neuroimmunol. 313:161–175. 29146293 10.1016/j.jneuroim.2017.11.003

[CIT0006] Dhivya Bharathi M, Justin-Thenmozhi A, Manivasagam T, Ahmad Rather M, Saravana Babu C, Mohamed Essa M, Guillemin GJ. 2019. Amelioration of aluminum maltolate-induced inflammation and endoplasmic reticulum stress-mediated apoptosis by tannoid principles of *Emblica officinalis* in neuronal cellular model. Neurotox Res. 35(2):318–330. 30242626 10.1007/s12640-018-9956-5

[CIT0007] Farzi MA, Sadigh-Eteghad S, Ebrahimi K, Talebi M. 2018. Exercise improves recognition memory and acetylcholinesterase activity in the beta amyloid-induced rat model of Alzheimer's disease. Ann Neurosci. 25(3):121–125. 30814820 10.1159/000488580 PMC6388429

[CIT0008] Gu L, Lu J, Li Q, Wu N, Zhang L, Li H, Xing W, Zhang X. 2020. A network-based analysis of key pharmacological pathways of *Andrographis paniculata* acting on Alzheimer’s disease and experimental validation. J Ethnopharmacol. 251:1–42. 10.1016/j.jep.2019.112488 31866509

[CIT0009] Gu L, Yu Q, Li Q, Zhang L, Lu H, Zhang X. 2018. Andrographolide protects PC12 cells against beta-amyloid-induced autophagy-associated cell death through activation of the Nrf2-mediated p62 signaling pathway. Int J Mol Sci. 19:1–15. 10.3390/ijms19092844 PMC6165383 30235892

[CIT0010] Gupta S, Mishra KP, Kumar B, Singh SB, Ganju L. 2020. Andrographolide attenuates complete Freund’s adjuvant induced arthritis via suppression of inflammatory mediators and pro-inflammatory cytokines. J Ethnopharmacol. 261:1–27. 10.1016/j.jep.2020.113022 32569719

[CIT0011] Huang Z, Ji H, Shi J, Zhu X, Zhi Z. 2020. Engeletin attenuates Aβ1-42-induced oxidative stress and neuroinflammation by Keap1/Nrf2 pathway. Inflammation. 43(5):1759–1771. 32445069 10.1007/s10753-020-01250-9

[CIT0012] Johnson VJ, Kim SH, Sharma RP. 2005. Aluminum-maltolate induces apoptosis and necrosis in neuro-2a cells: potential role for p53 signaling. Toxicol Sci. 83(2):329–339. 15537749 10.1093/toxsci/kfi028

[CIT0013] Komatsu M, Kurokawa H, Waguri S, Taguchi K, Kobayashi A, Ichimura Y, Sou YS, Ueno I, Sakamoto A, Tong KI, et al. 2010. The selective autophagy substrate p62 activates the stress responsive transcription factor Nrf2 through inactivation of Keap1. Nat Cell Biol. 12(3):213–223. 20173742 10.1038/ncb2021

[CIT0014] Lindsay CB, Zolezzi JM, Rivera DS, Cisternas P, Bozinovic F, Inestrosa NC. 2020. Andrographolide reduces neuroinflammation and oxidative stress in aged *Octodon degus*. Mol Neurobiol. 57(2):1131–1145. 31701436 10.1007/s12035-019-01784-6

[CIT0015] Lu J, Huang Q, Zhang D, Lan T, Zhang Y, Tang X, Xu P, Zhao D, Cong D, Zhao D, et al. 2020. The protective effect of DiDang tang against AlCl_3_-induced oxidative stress and apoptosis in PC12 cells through the activation of SIRT1-mediated Akt/Nrf2/HO-1 pathway. Front Pharmacol. 11:1–13. 32372957 10.3389/fphar.2020.00466 PMC7179660

[CIT0016] Peng H, Yang J, Li G, You Q, Han W, Li T, Gao D, Xie X, Lee BH, Du J, et al. 2017. Ubiquitylation of p62/sequestosome1 activates its autophagy receptor function and controls selective autophagy upon ubiquitin stress. Cell Res. 27(5):657–674. 28322253 10.1038/cr.2017.40 PMC5520855

[CIT0017] Peters F, Salihoglu H, Pratsch K, Herzog E, Pigoni M, Sgobio C, Lichtenthaler SF, Neumann U, Herms J. 2019. Tau deletion reduces plaque-associated BACE1 accumulation and decelerates plaque formation in a mouse model of Alzheimer’s disease. Embo J. 38(23):1–16. 10.15252/embj.2019102345 PMC6885735 31701556

[CIT0018] Santarino IB, Viegas MS, Domingues NS, Ribeiro AM, Soares MP, Vieira OV. 2017. Involvement of the p62/NRF2 signal transduction pathway on erythrophagocytosis. Sci Rep. 7(1):16. 28724916 10.1038/s41598-017-05687-1 PMC5517431

[CIT0019] Seo JY, Pyo E, An JP, Kim J, Sung SH, Oh WK. 2017. Andrographolide activates Keap1/Nrf2/ARE/HO-1 pathway in HT22 cells and suppresses microglial activation by Abeta42 through Nrf2-related inflammatory response. Mediators Inflamm. 2017:5906189– 5906113. 28373747 10.1155/2017/5906189 PMC5360972

[CIT0020] Song J. 2018. Animal model of aluminum-induced Alzheimer’s disease. Adv Exp Med Biol. 1091:113–127. 30315452 10.1007/978-981-13-1370-7_7

[CIT0021] Tian Y, Chang JC, Fan EY, Flajolet M, Greengard P. 2013. Adaptor complex AP2/PICALM, through interaction with LC3, targets Alzheimer’s APP-CTF for terminal degradation via autophagy. Proc Natl Acad Sci USA. 110(42):17071–17076. 24067654 10.1073/pnas.1315110110 PMC3801056

[CIT0022] Varela-Nallar L, Arredondo SB, Tapia-Rojas C, Hancke J, Inestrosa NC. 2015. Andrographolide stimulates neurogenesis in the adult hippocampus. Neural Plast. 2015:935403–935414. 26798521 10.1155/2015/935403 PMC4700200

[CIT0023] Wan Y, Liang Y, Liang F, Shen N, Shinozuka K, Yu JT, Ran C, Quan Q, Tanzi RE, Zhang C. 2019. A curcumin analog reduces levels of the Alzheimer’s disease-associated amyloid-β protein by modulating AβPP processing and autophagy. J Alzheimers Dis. 72(3):761–771. 31640096 10.3233/JAD-190562

[CIT0024] Wang N, Wang H, Li L, Li Y, Zhang R. 2019. beta-Asarone inhibits amyloid-beta by promoting autophagy in a cell model of Alzheimer’s disease. Front Pharmacol. 10:1–11. 32009952 10.3389/fphar.2019.01529 PMC6979317

[CIT0025] Wang S, Wu YY, Wang X, Shen P, Jia Q, Yu S, Wang Y, Li X, Chen W, Wang A, et al. 2020. Lycopene prevents carcinogen-induced cutaneous tumor by enhancing activation of the Nrf2 pathway through p62-triggered autophagic Keap1 degradation. Aging. 12(9):8167–8190. 32365333 10.18632/aging.103132 PMC7244072

[CIT0026] Wang W, Yang X, Chen Q, Guo M, Liu S, Liu J, Wang J, Huang F. 2020. Sinomenine attenuates septic-associated lung injury through the Nrf2-Keap1 and autophagy. J Pharm Pharmacol. 72(2):259–270. 31729764 10.1111/jphp.13202

[CIT0027] Xu Y, Tang D, Wang J, Wei H, Gao J. 2019. Neuroprotection of andrographolide against microglia-mediated inflammatory injury and oxidative damage in PC12 neurons. Neurochem Res. 44(11):2619–2630. 31562575 10.1007/s11064-019-02883-5

[CIT0028] Yang R, Liu S, Zhou J, Bu S, Zhang J. 2017. Andrographolide attenuates microglia-mediated Aβ neurotoxicity partially through inhibiting NF-κB and JNK MAPK signaling pathway. Immunopharmacol Immunotoxicol. 39(5):276–284. 28669260 10.1080/08923973.2017.1344989

[CIT0029] Yang Y, Willis TL, Button RW, Strang CJ, Fu Y, Wen X, Grayson PRC, Evans T, Sipthorpe RJ, Roberts SL, et al. 2019. Cytoplasmic DAXX drives SQSTM1/p62 phase condensation to activate Nrf2-mediated stress response. Nat Commun. 10(1):1–18. 31434890 10.1038/s41467-019-11671-2 PMC6704147

[CIT0030] Zhang Y, Wang G, Wang T, Cao W, Zhang L, Chen X. 2019. Nrf2-Keap1 pathway-mediated effects of resveratrol on oxidative stress and apoptosis in hydrogen peroxide-treated rheumatoid arthritis fibroblast-like synoviocytes. Ann NY Acad Sci. 1457(1):166–178. 31475364 10.1111/nyas.14196

